# Correlation analysis of quantitative MRI measurements of thigh muscles with histopathology in patients with idiopathic inflammatory myopathy

**DOI:** 10.1186/s41747-023-00350-z

**Published:** 2023-08-17

**Authors:** Fengdan Wang, Shiyuan Fang, Jia Li, Ling Yuan, Bo Hou, Jinxia Zhu, Yang Jiao, Zhi Liu, Min Qian, Francesco Santini, Qian Wang, Lin Chen, Feng Feng

**Affiliations:** 1 Department of Radiology, Peking Union Medical College Hospital, Chinese Academy of Medical Sciences and Peking Union Medical College, Beijing, China; 2 Department of Neurology, Peking Union Medical College Hospital, Chinese Academy of Medical Sciences and Peking Union Medical College, Beijing, China; 3 MR Collaboration, Siemens Healthineers Ltd., Beijing, China; 4 Department of General Internal Medicine, Peking Union Medical College Hospital, Chinese Academy of Medical Sciences and Peking Union Medical College, Beijing, China; 5 Department of Research and Analytic Services, University Hospital Basel, Basel, Switzerland; 6 Radiological Physics, University Hospital Basel, Basel, Switzerland; 7 Department of Biomedical Engineering, University of Basel, Allschwil, Switzerland; 8 Department of Rheumatology, Chinese Academy of Medical Sciences and Peking Union Medical College, Beijing, China

**Keywords:** Machine learning, Magnetic resonance imaging, Inflammation, Myositis, Thigh

## Abstract

**Objectives:**

To validate the correlation between histopathological findings and quantitative magnetic resonance imaging (qMRI) fat fraction (FF) and water T2 mapping in patients with idiopathic inflammatory myopathy (IIM).

**Methods:**

The study included 13 patients with histopathologically confirmed IIM who underwent dedicated thigh qMRI scanning within 1 month before open muscle biopsy. For the biopsied muscles, FF derived from the iterative decomposition of water and fat with echo asymmetry and least-squares estimation quantitation (IDEAL-IQ) and T2 time from T2 mapping with chemical shift selective fat saturation were measured using a machine learning software. Individual histochemical and immunohistochemical slides were evaluated using a 5-point Likert score. Inter-reader agreement and the correlation between qMRI markers and histopathological scores were analyzed.

**Results:**

Readers showed good to perfect agreement in qMRI measurements and most histopathological scores. FF of the biopsied muscles was positively correlated with the amount of fat in histopathological slides (*p* = 0.031). Prolonged T2 time was associated with the degree of variation in myofiber size, inflammatory cell infiltration, and amount of connective tissues (*p* ≤ 0.008 for all).

**Conclusions:**

Using the machine learning-based muscle segmentation method, a positive correlation was confirmed between qMRI biomarkers and histopathological findings of patients with IIM. This finding provides a basis for using qMRI as a non-invasive tool in the diagnostic workflow of IIM.

**Relevance statement:**

By using ML-based muscle segmentation, a correlation between qMRI biomarkers and histopathology was found in patients with IIM: qMRI is a potential non-invasive tool in this clinical setting.

**Key points:**

• Quantitative magnetic resonance imaging measurements using machine learning-based muscle segmentation have good consistency and reproductivity.

• Fat fraction of idiopathic inflammatory myopathy (IIM) correlated with the amount of fat at histopathology.

• Prolonged T2 time was associated with muscle inflammation in IIM.

**Graphical Abstract:**

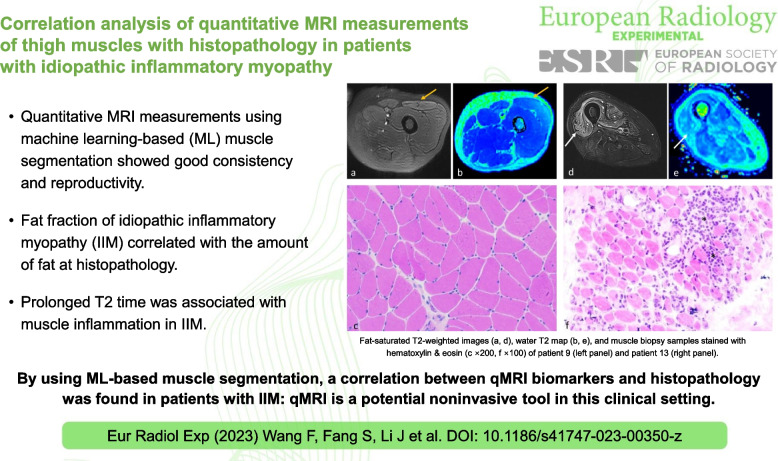

**Supplementary Information:**

The online version contains supplementary material available at 10.1186/s41747-023-00350-z.

## Background

Idiopathic inflammatory myopathy (IIM), including dermatomyositis, polymyositis, anti-synthetase syndrome, and immune-mediated necrotizing myopathy, is a group of diseases with progressive muscle weakness, which particularly affects the proximal lower extremities [1]. Skeletal muscle biopsy is the standard of care for the diagnosis and differentiation of IIM from hereditary myopathies [2]. Although different types of IIM may have unique histopathological characteristics, all IIM share similar histological features such as muscle fiber necrosis, degeneration, regeneration, and varying degrees of inflammatory cell infiltration in muscle tissues.

Unlike muscle biopsy, magnetic resonance imaging (MRI) can non-invasively assess large amounts of muscles (*e.g.*, both thighs). Qualitative conventional T1-weighted imaging can detect fatty infiltration in muscles reflecting chronic changes, while fat inversion-recovery or fat-suppressed T2-weighted imaging localizes muscle edema, thus indicating disease activity [3]. Muscle MRI is useful in determining whether a myopathy is active and in identifying the appropriate sites for biopsy [4]. Moreover, muscle MRI can be performed repeatedly during follow-up to monitor the treatment response, especially in IIM patients with normal serum creatine kinase levels.

To quantitatively evaluate muscle fatty infiltration and edema in IIM, some novel quantitative MRI (qMRI) sequences have been developed. An improved three-dimensional scanning Dixon-type sequence termed iterative decomposition of water and fat with echo asymmetry and least-squares estimation quantitation (IDEAL-IQ) can rapidly and accurately measure tissue fat fraction (FF) with good reproducibility [5, 6]. Muscle edema, resulting from increased intracellular or extracellular free water, is reflected by T2 prolongation measured by water T2 mapping [7, 8]. By using the newly designed machine learning (ML)-based muscle segmentation technique, FF and water T2 relaxation time can be more efficiently and accurately measured by avoiding sampling errors as compared to manual segmentation [9].

A correlation analysis between qMRI measurements and the histopathological findings of patients with IIM is essential for validating the application of qMRI as a non-invasive tool in the diagnostic workflow of this disease entity. However, previous studies on this topic are scarce and restricted by the use of only animal models [10, 11] or the heterogeneity of neuromuscular disorders [12, 13]. Therefore, the present study aimed to evaluate qMRI markers in patients with IIM by using muscle biopsy as the reference standard.

## Methods

### Patient population

**Fig. 1:**
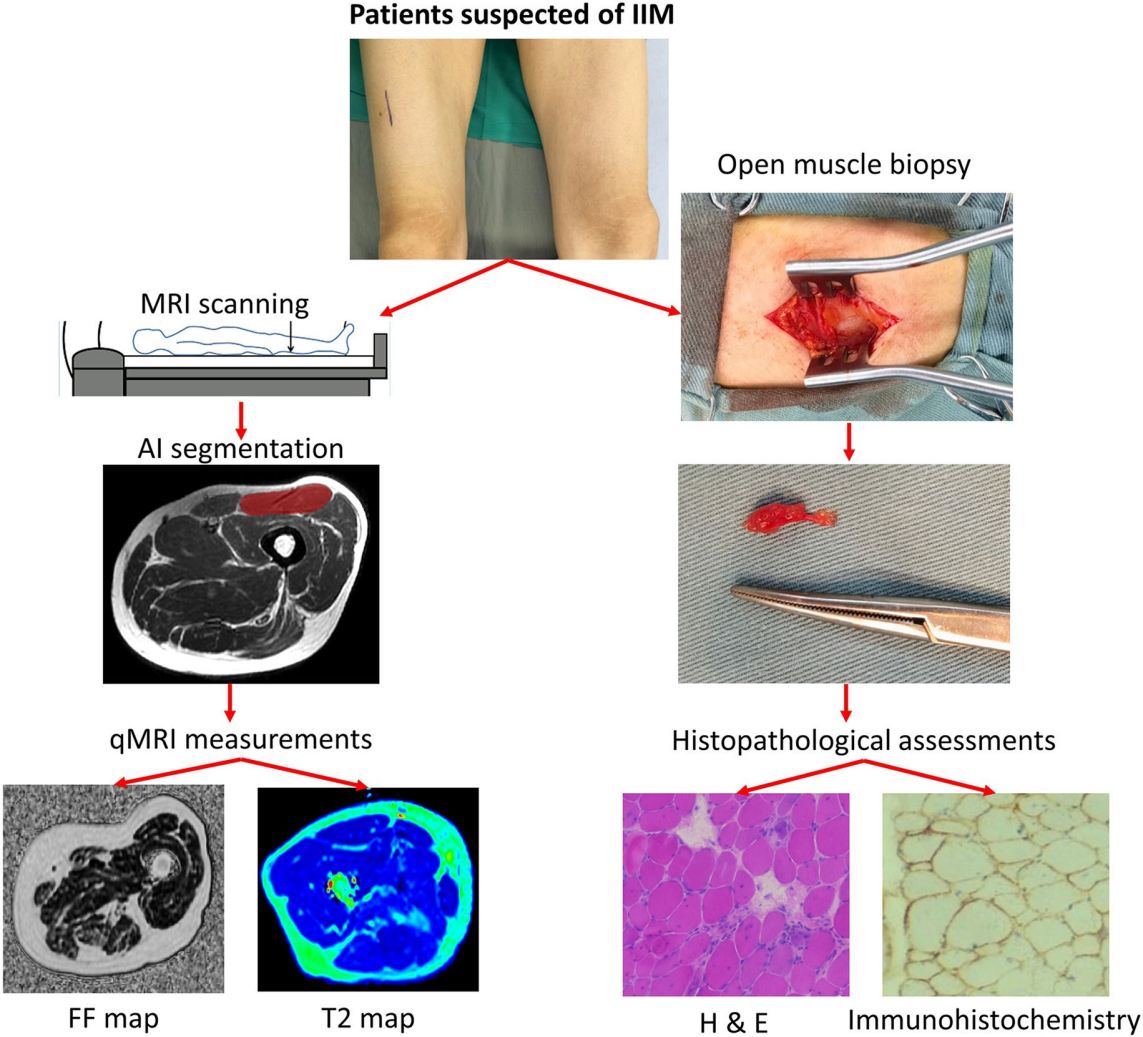
Study design of correlation analysis of quantitative magnetic resonance imaging (qMRI) measurements and histopathological findings in patients with idiopathic inflammatory myopathy. *IIM*, Idiopathic inflammatory myopathy; *H & E*, Hematoxylin and eosin

This single-center retrospective study was approved by the Institutional Review Board, and written informed consent was waived due to our retrospective nature. From January 2019 to July 2022, patients meeting the following criteria were included in the study: (1) patients suspected to have IIM and referred to the Department of Radiology for dedicated thigh qMRI scanning; (2) patients who underwent open skeletal muscle biopsy of thigh muscles within 1 month after MRI; and (3) patients diagnosed with IIM by rheumatologists according to the 2017 EULAR (European League Against Rheumatism/American College of Rheumatology) classification criteria [2] along with biopsy confirmation. The study design and flow chart are shown in Fig. 1.

### MRI protocol

Bilateral thigh muscles were scanned by a 3-T scanner (Discovery MR750w, General Electric Medical Systems, Milwaukee, WI, USA) with an 8-channel surface coil. The patients were placed in the feet-first supine position, and a 42-cm field of view (FOV) was positioned.

The structural MRI protocol included:

- coronal T1-weighted IDEAL sequence (repetition time (TR) 5.40 ms, echo time (TE) 2.54 ms, slice thickness 1 mm, gap 0 mm, and matrix 416 × 288);
- coronal T2-weighted IDEAL sequence (TR 4,750 ms, TE 114 ms, slice thickness 4 mm, gap = 4 mm, and matrix 448 × 256);
- Axial T2-weighted imaging with IDEAL (TR 5,250 ms, TE 120 ms, slice thickness 8 mm, gap 10 mm, and matrix 416 × 288).

The qMRI protocol included

- Axial IDEAL-IQ (TR 11.2 ms, TE, 1.5–9 ms, ΔTE 1.5 ms, slice thickness 6 mm, gap 3 mm, and matrix 320 × 192 interpolated to 512 × 512);
- Axial T2 mapping with chemical shift selective fat saturation (TR 1,046 ms, TE 7–56 ms, ΔTE 7 ms, slice thickness 6 mm, gap 7 mm, and matrix 320 × 160 interpolated to 512 × 512).

The total acquisition time was approximately 14 min and 30 s. After scanning, a set of 5-mm-thick axial T1-weighted images was reconstructed from the non-gap coronal T1-weighted images for clinical evaluation, and the FF map was generated from IDEAL-IQ automatically for FF measurements.

### qMRI measurements

Muscle segmentation and qMRI measurements were processed using an open-source software package termed “Deep Anatomical Federated Network” (DAFNE, https://dafne.network), which is based on a pretrained convolutional neural network and implements incremental and federated learning for continuous adaptation and improvement [14]. Axial T2-weighted images were used as the structural images for the automated segmentation of rectus femoris or vastus lateralis, which was biopsied after MRI. This segmentation was saved as masks, which were then imported and matched with the corresponding slices of FF and T2 maps by using the alignment module, thereby yielding muscle FF and T2 values of the biopsied rectus femoris in a fully automated manner.

A radiologist (L.Y., with 5 years of experience in general radiology, reader 1 (R1)) and a research physicist (F.S. who developed DAFNE, reader 2 (R2)) performed the segmentation and measurements independently. Both the readers were blinded to the scores of the histological and immunohistochemical assessments. If the inter-reader agreement was good, the average value of their measurements was used for correlation analysis. In addition, R2 measured FF and T2 values on the contralateral muscles in the same way as the biopsied side.

### Histological and immunohistochemical studies

Frozen muscle tissues were subjected to histological and immunohistochemical staining [15], including hematoxylin and eosin, modified Gomori trichrome, periodic acid Schiff, Oil Red O, nicotinamide adenine dehydrogenase, succinate dehydrogenase, cytochrome C oxidase, acid phosphatase (ACP), class I human major histocompatibility complex (MHC-1), membrane attack complex (C5b-9), CD4, CD8, CD20, and CD68.

### Histological and immunohistochemical assessments

The histological and immunohistochemical slides were evaluated by a visual analog scale. Two neuropathologists (J.L., reader 3 (R3) and M.Q., reader 4 (R4), with 5 and 15 years of work experience, respectively) were randomly and independently assigned to evaluate the histopathological alterations using a 5-point Likert score. Score 0 was normal, 1 indicated minor changes, 2 indicated mild changes, 3 indicated moderate changes, and 4 indicated severe changes.

The degree of variation of myofiber size, the amount of necrosis, inflammatory cell infiltration, and connective tissue, extent of fat infiltration, and ACP level were scored separately. The scoring of the inflammatory level was based on the levels of CD4, CD68, MHC-1, and complement components derived from the results of immunohistochemical staining. If the inter-reader agreement was good, the average value of their scores was used for correlation analysis.

### Statistical analysis

Data are reported as mean ± standard deviation unless differently specified. Intraclass correlation coefficient (ICC) was used to evaluate the inter-reader agreement of R1 and R2, and it was defined as good (ICC = 0.61–0.8) or perfect (ICC = 0.81–1.0). Cohen’s linearly weighted κ was used to evaluate the inter-reader agreement of R3 and R4, and it was defined as good (κ = 0.61–0.8) or perfect (κ = 0.81–1.0). Spearman correlation analysis was used to determine the correlation between qMRI and histopathological parameters: FF with scores of fat replacement; water T2 with scores of myofiber size variation and amount of necrotic fibers, inflammatory cells, connective tissue, ACP, CD4, CD68, MHC-1, and complement components. All data analyses were performed using SPSS v.26.0 software (SPSS Inc., Chicago, IL, USA) and MedCalc Statistical Software (version 20.09; MedCalc Software, Ostend, Belgium). A *p* value lower than 0.05 was considered statistically significant.

## Results

### Patient population

The study enrolled 13 participants, including 7 patients with dermatomyositis, 4 with polymyositis, 1 with anti-synthetase syndrome, and 1 with immune-mediated necrotizing myopathy. There were 8 males and 5 females (male-to-female ratio, 1.6), and the mean age was 43.2 years (range 19–72 years). The mean serum creatine kinase level was 2,600 U/L (range 17–10,378 U/L), and the mean interval between MRI and muscle biopsy was 14 days (range 3–30 days). The right vastus lateralis of 1 patient, the right rectus femoris of 2 patients, and the left rectus femoris of the other 10 patients were biopsied (Table 1).

**Table 1:** The clinical information of patients with idiopathic inflammatory myopathy

N	Sex	Age (years)	Chief complaint	Course of disease (months)	CK (24–195 U/L)	Electromyography	MRI to biopsy (days)	Biopsy site	Diagnosis
1	M	50	Myalgia, muscle weakness	2	2,146	Myogenic damage	23	Left RF	PM
2	M	51	Skin rash, muscle weakness, SOB	9	27	Myogenic damage	9	Right RF	DM
3	M	23	Myalgia, muscle weakness, dysphagia	2	8,225	Myogenic damage	25	Left RF	DM
4	M	15	Skin rash and muscle weakness	2	37	Myogenic damage	5	Left RF	DM
5	M	50	Myalgia, muscle weakness	96	1,761	-	4	Left RF	PM
6	F	24	Muscle weakness	1	7,792	Myogenic damage	25	Left RF	IMNM
7	F	72	Muscle weakness	1	256	Myogenic damage	30	Right RF	PM
8	F	25	Skin rash, muscle weakness	108	504	Myogenic damage	4	Left RF	ASS
9	M	67	Muscle weakness, SOB	1	17	Normal	8	Left RF	DM
10	F	66	Muscle weakness, SOB	4	26	Myogenic damage	3	Left RF	PM
11	M	32	Skin rash, muscle weakness	5	1,762	Myogenic damage	10	Left RF	DM
12	F	19	Skin rash, muscle weakness	2	10,378	-	29	Left RF	DM
13	M	65	Myalgia, muscle weakness	36	866	Myogenic damage	10	Right VL	DM

*ASS* Anti-synthetase syndrome, *CK* Creatine kinase, *DM* Dermatomyositis, *IMNM* Immune-mediated necrotizing myopathy, *PM* Polymyositis, *RF* Rectus femoris, *SOB* Shortness of breath, *VL* Vastus lateralis

### Inter-reader agreement analysis

By using DAFNE software, two readers segmented and measured the entire muscle that was biopsied. FF was calculated from the FF map generated by the IDEAL-IQ sequence, and water T2 was calculated from the T2 map with fat saturation. The FF of the biopsied muscles was 7.3% ± 5.8% and 7.3% ± 6.4% for R1 and R2, respectively, with an ICC of 0.934. The water T2 value was 53.8 ± 12.8 and 56.8 ± 16.1 ms for R1 and R2, respectively, with an ICC of 0.838.

The two neuropathologists, R3 and R4, reached an agreement regarding their ratings for myofiber size variation, the amount of inflammatory cells, and levels of CD4 and CD68 with κ values between 0.639 and 0.753, while regarding their ratings for the amount of fat tissue and connective tissues the κ values ranged from 0.841 to 0.927 (Supplementary Material, Table S1).

### Correlation between qMRI and histopathological parameters

**Fig. 2:**
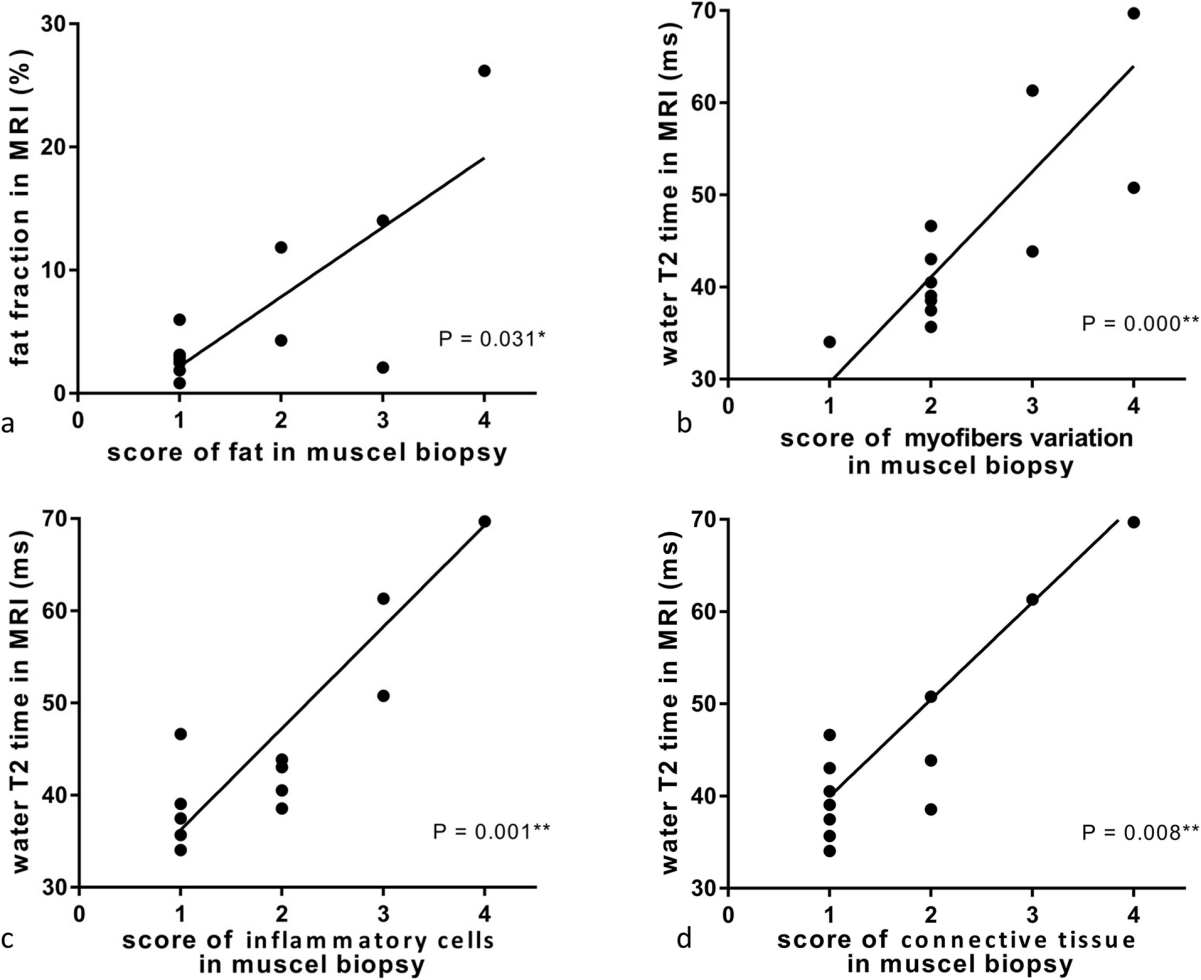
Correlations between quantitative magnetic resonance imaging parameters and histopathological findings of patients with idiopathic inflammatory myopathy

**Fig. 3:**
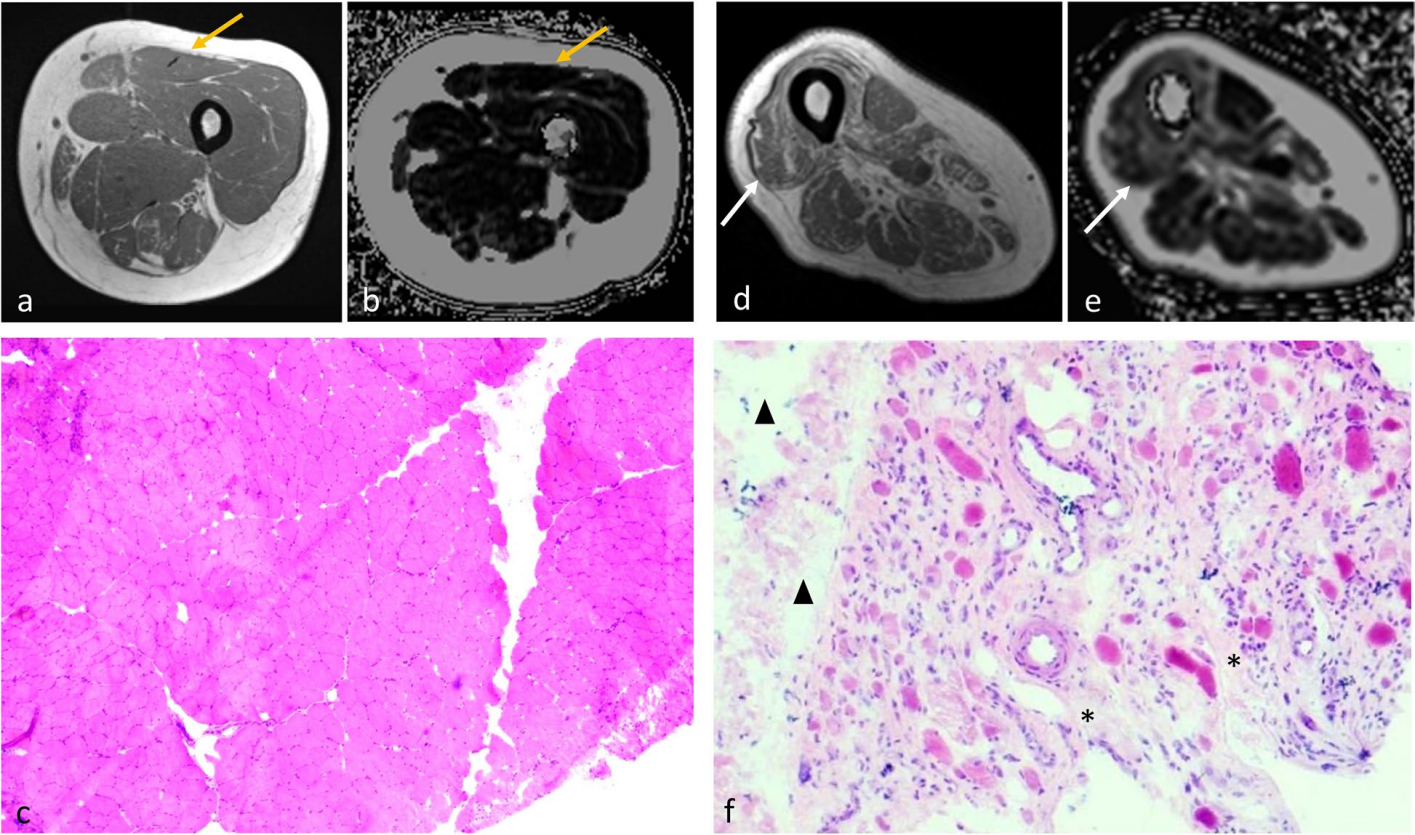
T1-weighted image (**a**, **d**), fat fraction (FF) map (**b**, **e**), and muscle biopsy samples stained with hematoxylin and eosin (**c** × 40, **f** × 40) of patient 8 (left panel) and patient 13 (right panel). Left rectus femoris (yellow arrows) of patient 8 was biopsied. The FF of this muscle was only 6.0% on the FF map; no fibrosis or fatty infiltrates were found in histopathology. Right vastus lateralis (white arrows) of patient 13 was biopsied. The FF of this muscle was as high as 26.1% on the FF map; pronounced fibrosis (stars) and fatty infiltrates (triangles) of the perineurium and endomysium were observed in histopathology

For the biopsied muscles, FF calculated from the IDEAL-IQ sequence was correlated with the score of fat tissue in the muscle samples. The average FF in the biopsied muscles was 7.3% ± 6.1% (range 0.8–26.2%), while the amount of fat in histopathological slides was 1.73 ± 1.12 (range 1–4); both these parameters were positively correlated (*r* = 0.597, *p* = 0.031) (Figs. 2a and 3).

**Fig. 4:**
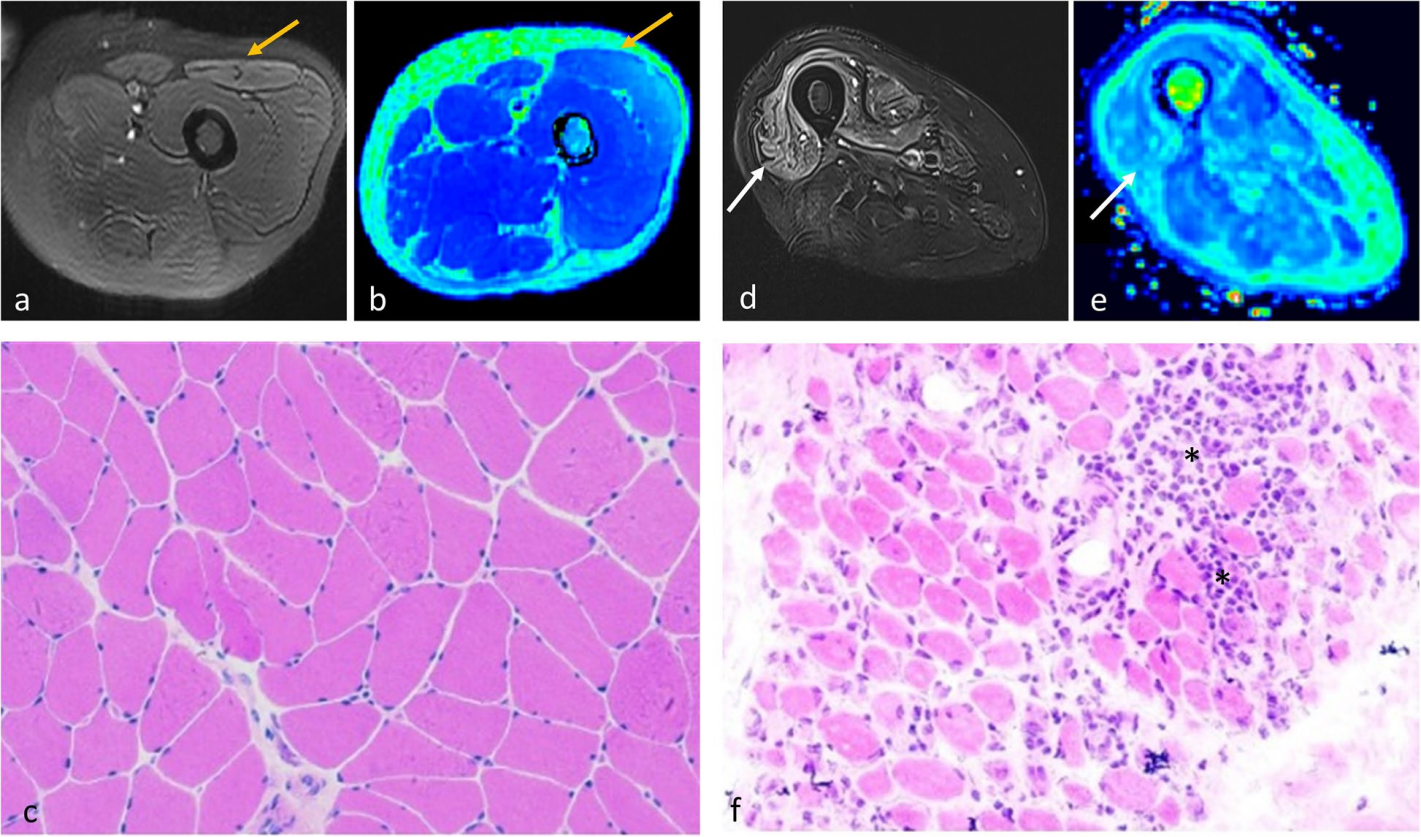
Fat-saturated T2-weighted images (**a**, **d**), water T2 map (**b**, **e**), and muscle biopsy samples stained with hematoxylin and eosin (**c** × 200, **f** × 100) of patient 9 (left panel) and patient 13 (right panel). Left rectus femoris (yellow arrows) of patient 9 was biopsied. The T2 value of this muscle was low at 34.1 ms. Mild variation in myofiber size was noted in histopathology, but no inflammatory cell infiltration or myofiber necrosis was observed. Right vastus lateralis (white arrows) of patient 13 was biopsied. The T2 value of this muscle was as high as 69.7 ms. Histopathological assessment showed marked perimysial and endomysial infiltration of inflammatory cells (stars) and myopathic changes, including myofiber size variation and myofiber necrosis

**Fig. 5:**
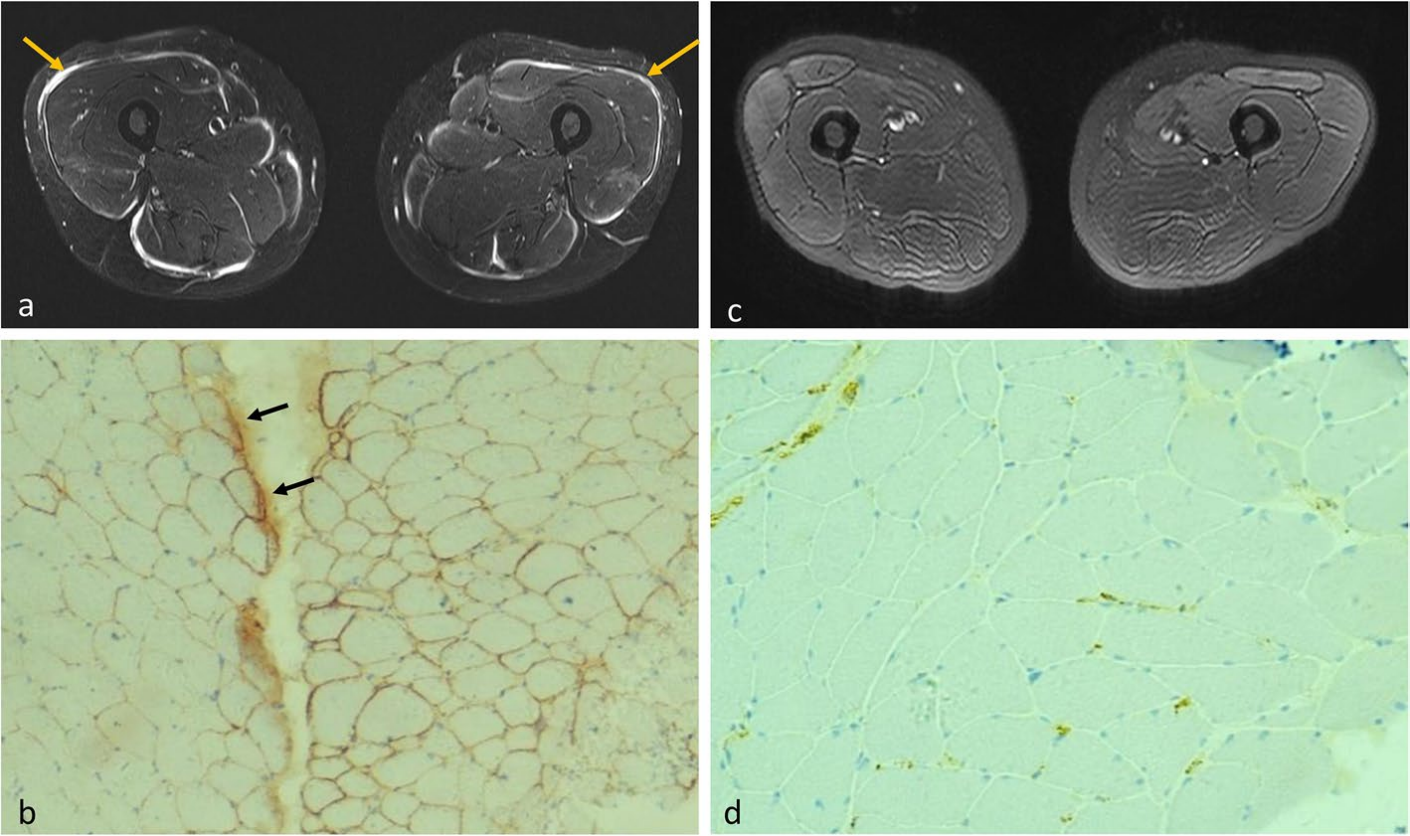
Fat-saturated T2-weighted images (**a**, **c**) and muscle biopsy samples subjected to immunohistochemical staining for membrane attack complex (C5b-9) (**b** × 100, **d** × 200) of patient 8 (left panel) and patient 9 (right panel). Both patients underwent biopsy of left rectus femoris. Patient 8 showed high-intensity myofascitis in bilateral muscles (yellow arrows) on fat-saturated T2-weighted images, while immunohistochemical staining showed prominent deposition of complement C5b-9, especially in the perimysium (black arrows). In contrast, patient 9 showed no such imaging findings or complement deposition

The correlation of water T2 resulting from increased free water with histopathological features was analyzed. Results showed that prolonged T2 time (54.7 ± 14.1 ms) was associated with the degree of myofiber size variation (2.38 ± 1.18), inflammatory cell infiltration (1.81 ± 1.42), and amount of connective tissues (1.69 ± 1.20) (*p* ≤ 0.008 (Figs. 2b–d and 4). However, the correlation between T2 value and other histopathological parameters, including myofiber necrosis and levels of ACP, CD4, CD68, MHC-1, and complement components, were statistically non-significant (*p* values from 0.063 to 0.522). Complement C5b-9 deposition was, however, found in patients with high-intensity myofascitis of bilateral muscles on fat-suppressed T2-weighted sequence (Fig. 5). These results demonstrate that water T2 reflects the degree of muscle inflammation and damage in patients with IIM.

For the contralateral muscles, FF and water T2 time was 7.5% ± 6.6% and 44.5 ± 4.4 ms, respectively, both of them showing no significant (*p* values from 0.190 to 0.981) association with the histopathological findings of the biopsied muscles during the correlation analysis.

## Discussion

In the present study, we analyzed 13 patients with muscle biopsy-confirmed IIM whose qMRI scans of bilateral thighs were performed within 1 month before biopsy of thigh muscles. Our results showed that by using ML-based segmentation, the measurements of muscle FF derived from IDEAL-IQ and water T2 time derived from T2 mapping have good consistency and reproductivity. By keeping muscle biopsy as the reference standard, the results for muscle qMRI parameters were consistent with the related histological features.

Muscle biopsy is the reference standard for IIM, particularly helpful when differentiating IIM from other neuromuscular disorders such as muscular dystrophy, inherited metabolic myopathies, and motor neuron diseases [16]. The disadvantages of muscle biopsy are that the procedure is invasive, the samples may not represent the complete picture of muscle involvement, and the technique is plagued with high false negative rate [17]. Thus, muscle biopsy may not be necessary in every patient with suspected IIM if the diagnosis can be established on the basis of clinical, laboratory, and MRI findings. Moreover, it is impractical to repeat muscle biopsy during follow-up to monitor the treatment response. In contrast, MRI has the advantages of non-invasiveness, coverage of large volumes of muscles, optimization of the biopsy site, and repeated performance at any time during the treatment course [18].

In MRI, FF (defined as the ratio of fat signal to the sum of signals from fat and water) can be quantified by magnetic resonance spectroscopy and extended Dixon’s methods [19]. The ^1^H spectrum of a muscle is dominated by lipid resonances, and the lipid peaks can be further subdivided into those arising from within (intramyocellular lipids) and between (extramyocellular lipids) muscle cells. For muscles, single-voxel techniques are generally preferred because of shimming difficulties over large volumes [20]. However, this limits its application in patients with IIM because fat infiltration is usually spatially heterogeneous.

By using a maximum likelihood method–echo symmetry and least squares, IDEAL-IQ is an extended Dixon type sequence, from which T2* decay can be incorporated into signal models [19]. Therefore, proton density FF maps with T2* decay correction are generated, which allow for large anatomical coverage with good accuracy and reproducibility [5]. Generated from IDEAL-IQ, FF measured by ML-based autosegmentation in our study was associated with fatty infiltration in patients with IIM. A previous study confirmed that FF calculated from IDEAL by manually delineating slice by slice agreed closely with the amount of fat tissue in muscle biopsy of patients with neuromuscular disorders [13]. Thus, this MRI-based biomarker is helpful to measure chronic damage–fatty replacement in patients with IIM.

In contrast, the active muscle inflammation of IIM could be quantitatively measured by the T2 map. Previous studies have shown that water T2 values derived from the T2 map of affected muscles in IIM were elevated as compared to those of volunteers [21], patients with inactive IIM [22], and those with unaffected muscles in IIM [23]. In addition, increased muscle T2 values were associated with visual inflammation scores [21, 24] and serum muscle enzymes [23]. A qMRI-histopathological correlation analysis found that water T2 time correlated with the amount of vacuolar alterations of myofibers and endomysial macrophages in skeletal muscle tissue; however, the study population of this correlation analysis was 10 patients with different neuromuscular disorders, and only one of the participants was diagnosed to have polymyositis [13]. In our present study (including only IIM patients), prolonged T2 time was associated with the degree of myofiber size variation, inflammatory cell infiltration, and amount of connective tissues. A borderline *p* value was also noted between T2 values and complement C5b-9 deposition. Therefore, increased T2 values measured from the water T2 map, representing increased intracellular and extracellular free water, may be a cumulative figure of various pathological changes of IIM.

To extract these MRI-based biomarkers (FF and water T2 value) from muscles, the traditional method involves drawing a region of interest manually; however, this process is time-consuming (several hours per subject), requires dedicated work of experienced operators, and is associated with selection errors [25]. Though deep learning methods are very scarcely used in neuromuscular disorders as compared to that in other scientific fields, these methods hold promise in muscle segmentation of MR images. In our present study, we used the automatic segmentation tool DAFNE based on customized versions of the VNet and ResNet architectures, and it showed good agreement with manually segmented labeled images to create muscle segmentation [14]. The inter-reader agreement of measurements was good using DAFNE. We also found that water T2 mapping of thigh muscles of 64 patients with IIM segmented by this software could detect muscle inflammation even in patients with normal serum levels of creatine kinase [9].

With the advent of ML-based segmentation, qMRI techniques such as IDEAL-IQ and T2 mapping could provide quantitative measurements more accurately, efficiently, and cost-effectively, holding promise in the diagnostic workflow of IIM and other myopathies. The quantification of FF, a more precise method than semi-quantitatively scoring conventional structured MR images, correlates well with histology and clinical function [26], thus could be used to evaluate the stage of diseases, and as an endpoint to monitor the treatment efficacy [27]. Additionally, water T2 values, a measurement of disease activity, represents the degree of inflammation and treatable target in IIM, which could serve as a biomarker to accurately assess the treatment response to glucocorticoid, immunosuppressive or biological agents [28].

The limitations of our present study must be acknowledged. First, the number of patients is relatively small since muscle biopsy was performed only in patients presenting with clinical and/or laboratory evidence of myopathy and who did not show extramuscular manifestations such as a typical dermatomyositis rash or a myositis-specific autoantibody. Nevertheless, this was the largest group of patients with IIM for qMRI and histopathology correlation study. However, we admitted that there is a concentration of data points in a single score of pathological findings, which may lead to false positives. The inter-reader agreement for MHC-1 levels was slight (κ = 0.128), while that for myofiber necrosis, levels of ACP, and complement components were moderate (κ = 0.444–0.589). Second, this was a retrospective study in which qMRI was measured for the entire muscle that was biopsied, but only a small section of the muscle was removed for histopathological analysis. Therefore, sampling error should be considered as a confounder in this study. In addition, the present study did not directly compare the ML-based whole muscle segmentation and manually assigning ROIs with being fitted to the biopsy site, because only patients who underwent open skeletal muscle biopsy after MRI examination were included, and thus we were not able to define the accurate slices of biopsy site when performing qMRI measurements. Third, muscle edema and fatty replacement in IIM were heterogenous, which implies that qMRI markers (FF and water T2 value) of the biopsied muscle (vastus lateralis and rectus femoris in this study) did not represent the involvement of the entire skeletal muscle of the patients. Proximal skeletal muscle weakness, especially in lower extremities, is usually the primary complaint of patients with IIM; hence, the usual muscle targets for biopsy are the quadriceps in the thighs.

In conclusion, by using ML-based muscle segmentation, we found a positive correlation between qMRI biomarkers and histopathological findings in patients with IIM. The results of this study provide a basis for using qMRI as a non-invasive tool in the clinical practice of this disease entity.

### Supplementary Information

**Additional file 1:** **Table S1.** Inter-observer agreements of semi-quantify histopathological findings.

## Data Availability

The anonymized dataset supporting the conclusions of this article is available upon request.

## Abbreviations

ACP: Acid phosphatase
DAFNE: Deep Anatomical Federated Network
FF: Fat fraction
ICC: Intraclass correlation coefficient
IDEAL-IQ: Iterative decomposition of water and fat with echo asymmetry and least-squares estimation quantitation
IIM: Idiopathic inflammatory myopathy
MHC-1: Class I human major histocompatibility complex
ML: Machine learning
MRI: Magnetic resonance imaging
qMRI: Quantitative magnetic resonance imaging
R1: Reader 1
R2: Reader 2
R3: Reader 3
R4: Reader 4
TE: Echo time
TR: Repetition time
