# Supplementary material for: Correlation analysis of quantitative MRI measurements of thigh muscles with histopathology in patients with idiopathic inflammatory myopathy
Source: Eur Radiol Exp. 2023 Aug 17;7:51. doi: 10.1186/s41747-023-00350-z (PMC10435435; doi:10.1186/s41747-023-00350-z)
Supplement: Supplementary file 1 — Additional file 1: Table S1. Inter-observer agreements of semi-quantify histopathological findings. [file 41747_2023_350_MOESM1_ESM.pdf]

Correlation analysis of quantitative MRI measurements of thigh muscles with histopathology in patients with idiopathic inflammatory myopathy

Table S1. Inter-observer agreements of semi-quantify histopathological findings.

	Fat tissue	Myofiber size variation	Myofiber necrosis	Inflammatory cells	Connective tissues	ACP	CD4	CD68	MHC-1	Complement components
**Reader 3**	1.77 ± 1.24	2.46 ± 1.20	1.77 ± 1.09	1.85 ± 1.57	1.77 ± 1.17	1.85 ± 1.28	1.30 ± 0.82	2.50 ±0.85	2.30 ± 1.70	1.50 ± 1.27
**Reader 4**	1.69 ± 1.03	2.31 ± 1.25	1.62 ± 1.04	1.77 ± 1.46	1.62 ± 1.26	1.62 ± 1.19	1.10 ±0.74	2.20 ± 0.79	2.40 ± 1.43	1.20 ± 1.14
**κ value**	0.927	0.639	0.589	0.753	0.841	0.491	0.744	0.651	0.128	0.444
